# 384. SARS-CoV-2 Surveillance Testing Patterns among Hospitalized Pediatric Patients in a Single Academic Medical Center

**DOI:** 10.1093/ofid/ofab466.585

**Published:** 2021-12-04

**Authors:** Areej Bukhari, Jessica Seidelman, Becky A Smith, Sarah S Lewis, Michael J Smith, Rebekah W Moehring, Deverick J Anderson, Ibukunoluwa Akinboyo

**Affiliations:** 1 Duke University, Durham, North Carolina; 2 Duke University Medical Center, Durham, North Carolina; 3 Duke Center for Antimicrobial Stewardship and Infection Prevention, Durham, NC

## Abstract

**Background:**

Children infected with SARS-CoV-2 often have mild or no symptoms, making symptom screening an ineffective tool for determining isolation precautions. As an infection control measure, universal pre-procedural and admission SARS-CoV-2 testing for pediatric patients was implemented in April and August 2020, respectively. Limited data exist on the utility screening programs in the pediatric population.

**Methods:**

We performed a retrospective cohort study of pediatric patients (birth to 18 years) admitted to a tertiary care academic medical center from April 2020 to May 2021 that had one or more SARS-CoV-2 point-of-care or polymerase chain reaction tests performed. We describe demographic data, positivity rates and repeat testing trends observed in our cohort.

**Results:**

A total of 2,579 SARS-CoV-2 tests were performed among 1,027 pediatric inpatients. Of these, 51 tests (2%) from 45 patients (4.3%) resulted positive. Community infection rates ranged from 4.5-60 cases/100,000 persons/day during the study period. Hispanic patients comprised 16% of the total children tested, but were disproportionately overrepresented (40%) among those testing positive (Figure1). Of 654 children with repeated tests, 7 (0.1%) converted to positive from a prior negative result. Median days between repeat tests was 12 (IQR 6-45), not necessarily performed during the same hospital stay. Five of these 7 patients had tests repeated < 3 days from a negative result, of which only 2 had no history of recent infection by testing performed at an outside facility. Pre-procedural tests accounted for 35% of repeat testing, of which 0.9% were positive. Repeated tests were most frequently ordered for patients in hematology/oncology (35%) and solid organ transplant/surgical (33%) wards, each with < 3% positive conversion rate. Notably, no hematopoietic stem cell transplant patients tested positive for SARS-CoV-2 during the study period.

Pediatric SARS-CoV-2 Testing Distributed by Race/Ethnicity

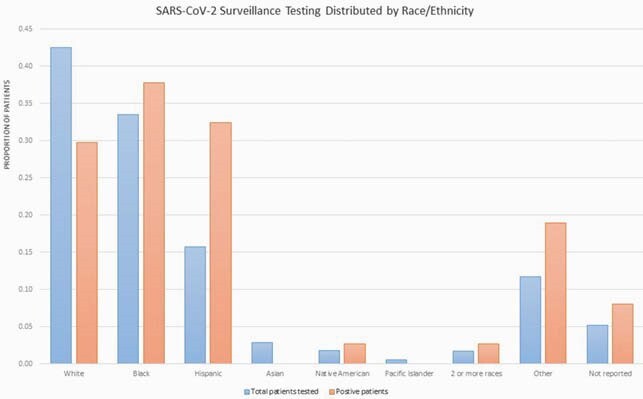

**Conclusion:**

The positivity rate of universal pre-procedural and admission SARS-CoV-2 testing in pediatric patients was low in our inpatient cohort. Tests repeated < 3 days from a negative result were especially low yield, suggesting limited utility of this practice. Diagnostic testing stewardship in certain populations may be useful, especially as community infection rates decline.

**Disclosures:**

**Michael J. Smith, MD, M.S.C.E**, **Merck** (Grant/Research Support)**Pfizer** (Grant/Research Support) **Rebekah W. Moehring, MD, MPH**, **UpToDate, Inc.** (Other Financial or Material Support, Author Royalties)

